# Sero-prevalence, risk factors and distribution of sheep and goat pox in Amhara Region, Ethiopia

**DOI:** 10.1186/s12917-017-1312-0

**Published:** 2017-12-11

**Authors:** Tsegaw Fentie, Nigusie Fenta, Samson Leta, Wassie Molla, Birhanu Ayele, Yechale Teshome, Seleshe Nigatu, Ashenafi Assefa

**Affiliations:** 1 College of Veterinary Medicine and Animal Sciences, University of Gondar, P. O. Box 196, Gondar, Ethiopia; 2 Livestock and Fisheries Development Office, Dembia District, North Gondar, Ethiopia; 3 College of Veterinary Medicine and Agriculture, Addis Ababa University, P. O. Box 34, Bishoftu, Ethiopia; 4 Faculty of Agriculture, Debre Markos University, Debre Markos, Ethiopia

**Keywords:** Amhara region, Sero-prevalence, Sheep and goat pox, Ethiopia

## Abstract

**Background:**

Sheep pox and goat pox are contagious viral diseases of sheep and goats, respectively. The diseases result in substantial economic losses due to decreased milk and meat production, damage to hides and wool, and possible trade restriction. A study was undertaken in Amhara region of Ethiopia. A cross-sectional study design was used to estimate the sero-prevalence and identify associated risk factors, while retrospective study design was used to assess the temporal and spatial distribution of the disease. A total of 672 serum samples were collected from 30 Kebeles and tested using virus neutralization test.

**Results:**

From a total of 672 sera tested, 104 (15.5%) were positive for sheep and goat pox virus antibody; from which 56 (17%) were sheep and 48 (14%) were goats. The diseases were prevalent in all study zones, the highest sero-prevalence was observed in South Gondar (20.9%) and the lowest in North Gondar and West Gojjam zones (11.9% each). From the potential risk factors considered (species, sex, age, agro-ecology and location); only sex and age were significantly associated (*p* < 0.05) with the diseases in multivariable logistic regression. Female and young animals were at higher risk than their counterparts. From January 2010 to December 2014, a total of 366 outbreaks, 12,822 cases and 1480 deaths due to SP and 182 outbreaks, 10,066 cases and 997 deaths due to GP were recorded in Amhara National Regional State.

**Conclusion:**

Both the serological and the outbreak data revealed that sheep and goat pox is one of the most prevalent and widespread diseases of sheep and goats in the study area. Hence, annual mass vaccination program must be implemented for economic and viable control of sheep and goat pox diseases in the Amhara region in particular and at a national level in general.

**Electronic supplementary material:**

The online version of this article (10.1186/s12917-017-1312-0) contains supplementary material, which is available to authorized users.

## Background

Estimates indicate that about 25.5 million sheep and 24.1 million goats are reared in the sedentary areas of Ethiopia excluding the non-sedentary population of three zones of Afar and six zones of Somali region. This makes Ethiopia be home to one of the largest heads of small ruminants in Africa after Nigeria [1, 2]. However, studies indicate that the current contributions of the livestock subsector which includes small ruminant production to the national economy at either the macro- or micro-level to be limited and below the potential [3].

Infectious diseases are amongst the major factors which limit the production and productivity of small ruminant; sheep pox (SP) and goat pox (GP) being topping the list [4]. Sheep pox and GP are viral diseases of sheep and goats characterized by fever, pyrexia and generalized skin lesions [5]. In susceptible herds, morbidity is 75-100% and case fatality, depending on the virulence of the virus is between 10 and 85% (19). The viruses causing these diseases are members of the genus *Capripoxvirus,* subfamily *Chordopoxvirinae* and family *Poxviridae*. There is close genetic relatedness of these *Capripoxvirus* isolates. The diseases induced by strains of SP virus, GP virus and lumpy skin disease virus cannot be differentiated clinically and serologically, including virus neutralization test. However, distinct host preferences exist with most strains of SP virus and GP virus causing more severe disease in the homologous host [6, 7].

The geographical distribution of SP and GP have been shown to extends from Africa, north of the equator to the Middle East and Asia including the former Soviet Union, India and China [4]. Many authors have reported the occurrence of SP and GP from east African countries namely Sudan and Kenya [8–11]. SP and GP are among the most important diseases of sheep and goats in Ethiopia following Peste des petits ruminants (PPR) and contagious caprine pleuropneumonia (CCPP). Questionnaire surveys and case reports indicate an occurrence of the diseases in Ethiopia including Amhara National Regional State (ANRS) [12, 13]. A recent study by Gelaye et al.*,* [14], indicated GP and lumpy skin disease virus to be responsible for the Capripox outbreaks in small ruminants and cattle in different parts of Ethiopia.

Sheep pox and GP are major constraints to trade and introduction of exotic breeds of sheep and goats; hindering efforts to improve local sheep and goats through importation of improved breeds [15]. The diseases are associated with significant production losses because of reduced milk yield, decreased weight gain, increased abortion rates, damage to wool and hides, and increased susceptibility to pneumonia and fly strike, while also being a direct cause of mortality [16].

Despite its considerable economic importance and threats to trade, information on the sero-prevalence and distribution of the diseases in Ethiopia in general and Amhara region, in particular, is absent. A better understanding of its prevalence and distribution would lead to improved disease control measures. Therefore, this study was aimed to estimate the sero-prevalence, assess risk factors and distribution of sheep and goat pox in ANRS.

## Methods

### Study area

**Fig. 1:**
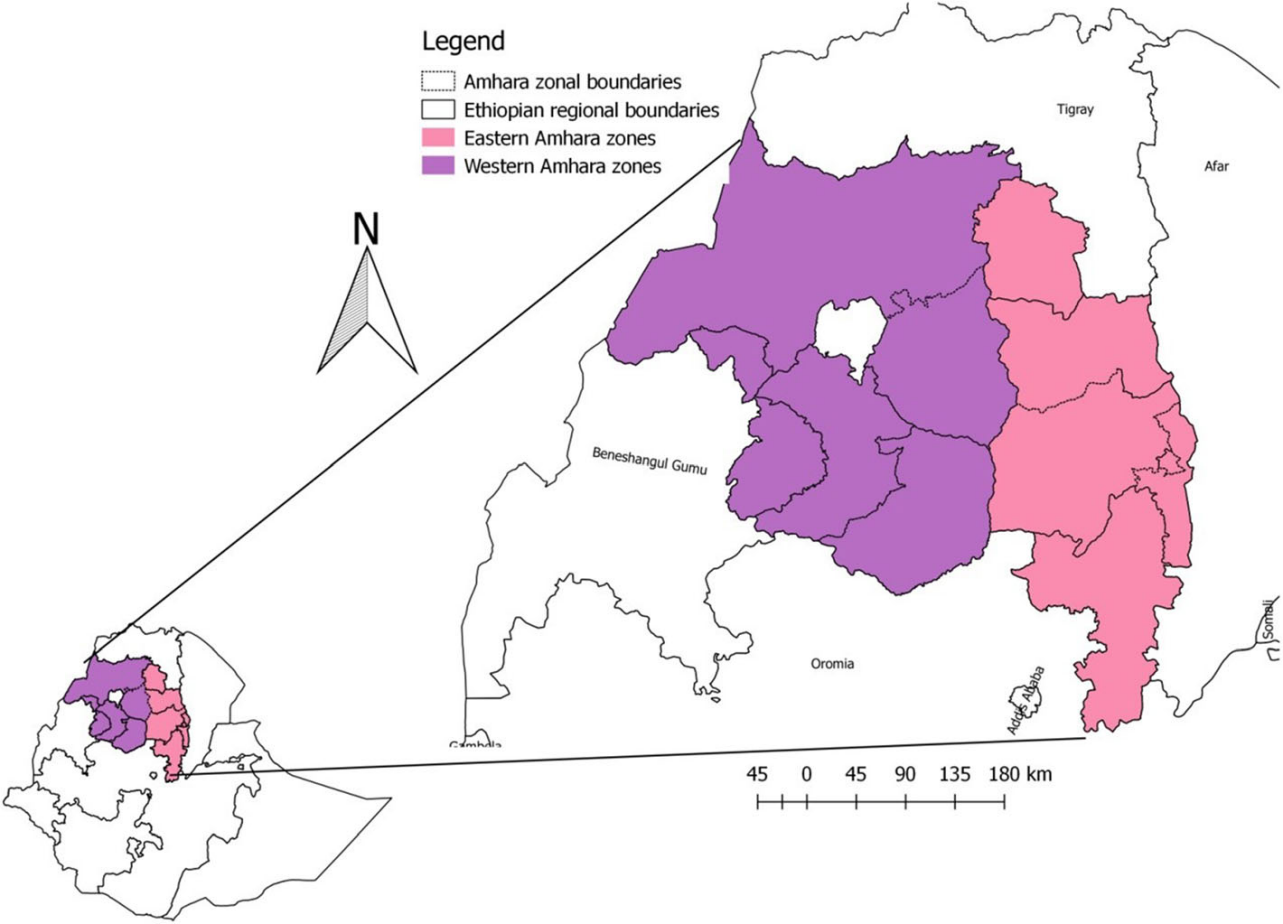
Map of the study area

The study has two components: sero-surveillance and retrospective studies. The sero-surveillance study was conducted in western part of the Amhara Regional National State (ANRS) whereas the retrospective study was undertaken in whole ANRS. The ANRS is located in the north western part of Ethiopia between 9°20′ and 14°20′ North latitude and 36° 20′ and 40° 20′ East longitude. The region borders with Tigray in the North, Afar in the East, Oromia in the South and Benishangul-Gumuz in the Southwest and the country of Sudan in the West (Fig. 1). The study area has diverse agro-climatic conditions; ranging from hot lowlands to cold highlands. Areas less than 1500 m.a.s.l. were considered as lowland, areas ranging from 1500 to 2500 m.a.s.l. were considered as midland and areas greater than 2500 m.a.s.l. were considered as highland. Western Amhara region has five zones (North Gondar, South Gondar, East Gojjam, West Gojjam and Awi). There are 66 districts in the five zones.

### Study population and sampling strategy

An estimated 6.6 million sheep and goats are reared in western Amhara region [1]. Sheep and goats that were kept under the extensive farming system, aged greater than 5 months were considered for the sero-surveillance study. Information about Capripox vaccination practice was collected from district animal husbandry departments and livestock owners. Kebeles/localities free of capripox vaccination in the past 1 year were included in the study. From 5 zones, districts and Kebeles were selected based on their agro-climatic zones and history of Capripox vaccination. A total of 10 districts, proportionally from each administrative zone (3 from North Gondar, 2 from South Gondar, 2 from West Gojjam, 2 from East Gojjam and 1 from Awi zone) were selected. Two districts were selected from highland, three districts from midland and five districts from lowland agro-ecology. Thirty Kebeles from ten selected districts; five Kebeles from highland, nine Kebeles from midland and 16 Kebeles from lowland were considered for the cross-sectional study. Systematic random sampling technique was used to sample individual animals in selected Kebeles.

### Study design

Cross-sectional and retrospective study designs were employed in this study. Cross-sectional study design was used to estimate the sero-prevalence of SP and GP and retrospective study design was used to assess their temporal and spatial distribution.

### Sample size determination

The sample size was calculated according to the formula given by Thrusfield [17], using 50% expected prevalence (since there is no previous prevalence report from the study area), 5% desired absolute precision and 95% confidence level.

The formula used:

$$ n=\frac{z^{2\ast } pq}{d^2} $$

Where: z = 1.96, *p* = 0.5, q = 1-p and d = 0.05 (the desired level of precession). The calculated sample size became 384. To increase the precision, the sample size was almost doubled to a total of 672 (329 sheep and 343 goats).

Due to the difference in population size in the 5 zones, 10 districts and even within Kebeles in the same district, sample size was allocated proportionally based on the existing sheep and goat population per zones/districts. Hence 22.47% (*n* = 151) of the sample was drawn from North Gondar zone, 22.77% (*n* = 153) from South Gondar zone, 22.47% (*n* = 151) from West Gojjam zone, 16.96% (*n* = 114) from East Gojjam zone and 15.33% (*n* = 103) from Awi zone.

### Data collection and laboratory analysis

Whole blood was collected from the jugular vein of sheep and goats using plain 10 ml vacutainer tubes and 19 gauge sterile needles. The samples were labelled to allow identification of each animal. Additional file 1 on potential risk factors (such as species, age, and sex) was recorded during sampling. The samples were kept in slant position overnight to allow serum separation. Serum was decanted and aliquoted into cryovials and stored in a freezer (-20 °C) at University of Gondar laboratory before transported to National Veterinary Institute (NVI). The serum samples were tested for the presence of sheep and goat pox antibodies using virus neutralization test (VNT) in NVI virology laboratory following the procedures described by Boshra et al. [18]. Sheep pox and GP antibodies cannot be distinguished by VNT, thus, it is called ‘sheep and goat pox antibody’ when referring to the serological result. Information regarding the age of the animal was obtained from the owner of the animal and classified as young (5 months to ≤1.5 years) and adults (> 1.5 years).

Five years (January 2010 to December 2014) retrospective data on SP and GP outbreak was also compiled. The data was obtained from the Epidemiology Unit of Federal Ministry of Livestock and Fisheries (MoLR). The retrospective epidemiological data contain the number of SP and GP outbreaks, sick and deaths, the population at risk and the species involved. The data were extracted from district monthly reports. Sheep pox and GP cases were identified by veterinary clinical diagnosis.

### Data management and analysis

The collected data was classified; filtered, coded using Microsoft Excel spreadsheet (Additional file 1) and analyzed by using STATA version 12. Descriptive statistics were used to present the results. Univariable and multivariable logistic regression analyses were used to identify the risk factors associated with Sheep and goat pox. Likelihood ratio test was used to determine the variables in the final multivariable logistic regression model. Backward variable illumination procedure was employed by using *p* = 0.05 as a cut-off value. In all analysis, the result is considered statistical significance if *p* < 0.05. QGIS v 2.18.0 was used to map the distribution of SP and GP in ANRS.

## Results

### Sero-prevalence and risk factors

**Fig. 2:**
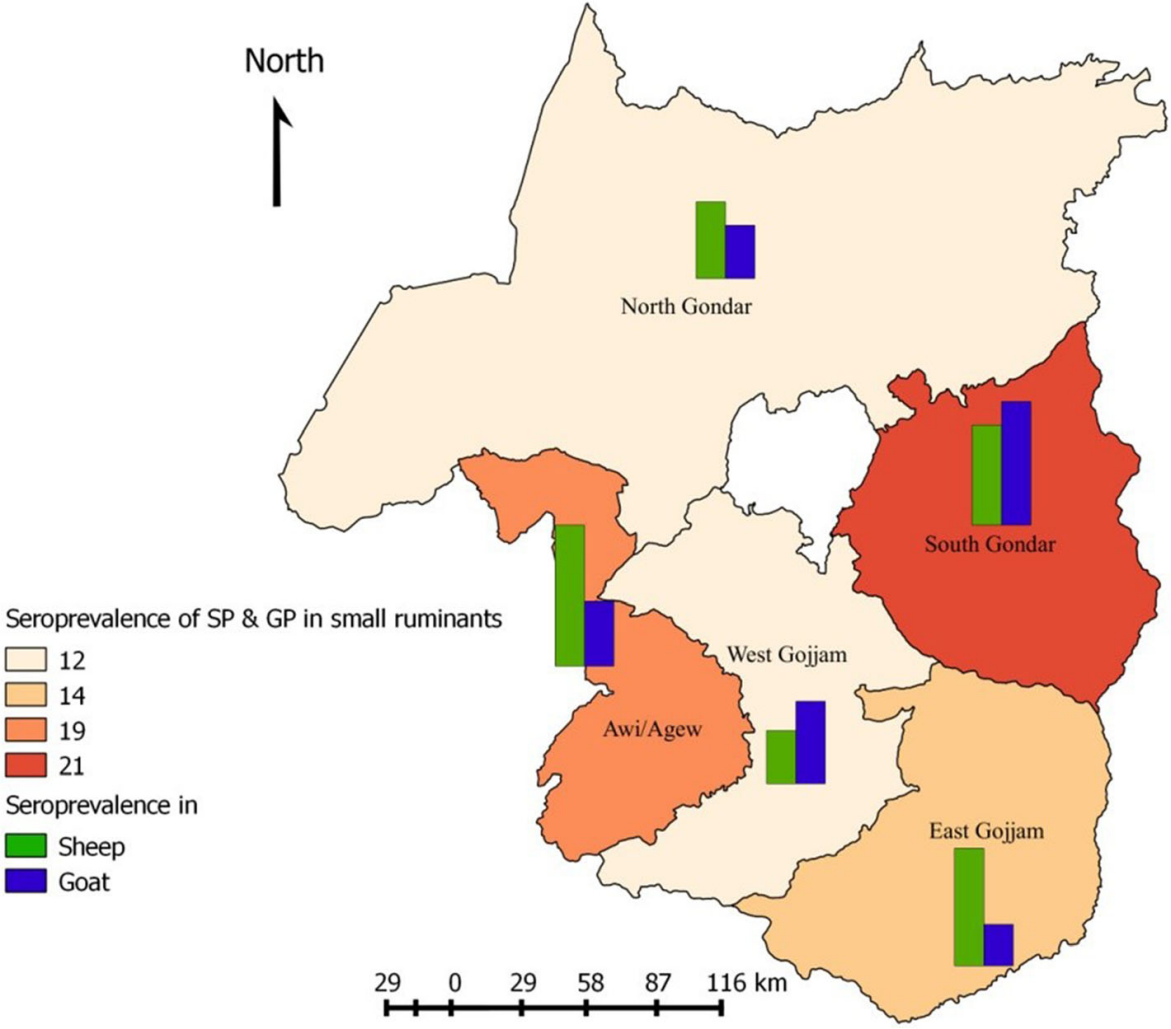
The sero-prevalence of SP and GP in western Amhara zones

Serum samples of both sheep and goat from all the five zones of western Amhara region were evaluated for previous exposure to sheep and goat poxviruses using virus neutralization test (VNT). Of the total 672 (329 sheep and 343 goats) blood sera tested, 104 (56 sheep and 48 goats) samples were positive for sheep and goat pox antibody. The overall sero-prevalence of sheep and goat pox was 15.5% (95% CI: 12.7, 18.2) in which 17% (95% CI: 12.9, 21.1) in sheep and 14% (95% CI: 10.3, 17.7) in goats. However, the antibody prevalence variation was not statistically significant between sheep and goats (*P* > 0.05). All the studied zones had small ruminants that were sero-positive for sheep and goat pox antibody. The highest sero-prevalence was observed in South Gondar (20.9%) followed by Awi (19.4%) (Fig. 2).

The univariable logistic regression analysis indicates that the sero-positivity varies among different localities, the sero-positivity in South Gondar being significantly higher than North Gondar (*p* < 0.05). Animals from all the study districts were positive for sheep and goat pox viruses antibody; the highest sero-prevalence was recorded in Fogera (27.3%), followed by Guangua (19.4%) and Debre-Elias (18.3%). The sero-prevalence of sheep and goat pox in Fogera is significant (*p* < 0.05) higher than Gonder zuria, West Belesa, Mecha, Javitehinan, and Gozamin. The antibody prevalence was not varied significantly among the agro-ecologies (Table 1).

**Table 1:** Univariable analysis of risk factors for sheep and goat pox antibodies categorized by location

Variables		Number sampled	Prevalence (%)	Odds ratio	Confidence interval	*P* value
Zone	North Gondar	151	18(11.9)	Ref	–	–
	West Gojjam	151	18(11.9)	1.0	0.49-2.0	1.0
	South Gondar	153	32(20.9)	1.9	1.04-3.66	0.036
	Awi	103	20(19.4)	1.8	0.89-3.56	0.103
	East Gojjam	114	16(14.03)	1.2	0.59-2.48	0.611
District	Fogera	66	18(27.3)	Ref	–	–
	Simada	87	14(16.1)	0.51	0.23- 1.12	0.095
	Gondar zuria	69	7(10.1)	0.30	0.12-0.78	0.013
	West Belesa	40	4(10)	0.30	0.09-0.95	0.041
	Dabat	42	7(16.6)	0.53	0.20-1.41	0.207
	Mecha	81	10(12.4)	0.38	0.16-0.88	0.025
	Javitehinan	70	8(13.4)	0.34	0.14-0.86	0.022
	Gozamin	54	5(9.3)	0.27	0.09-0.79	0.017
	Debre Elias	60	11(18.3)	0.60	0.26-1.39	0.236
	Guangua	103	20(19.4)	0.64	0.31-1.33	0.235
Agro-ecology	Highland	96	12(12.5)	Ref	–	–
	Midland	199	33(16.6)	1.4	0.68-2.83	0.362
	Lowland	377	59(15.7)	1.3	0.67-2.53	0.441

The host related risk factors; species, sex, and age were also analysed using univariable logistic regression. As shown in Table 2, the sero-prevalence of sheep and goat pox varies significantly between male and female animals as well as among different age categories. The odds of sero-positivity in female animals and young age animals were 1.75 and 2.2 times higher than male and adult animals, respectively.

**Table 2:** Univariable analysis of risk factors for sheep and goat pox categorized by animal factor

Variables		Number sampled	Prevalence (%)	Odds ratio	Confidence interval	*P* value
Species	Goat	343	48(14.0)	Ref	–	–
	Sheep	329	56(17.0)	1.26	0.83-1.92	0.279
Sex	Male	251	28(11.2)	Ref	–	–
	Female	421	76(18.1)	1.75	1.10-2.79	0.018
Age	Adult	444	52(11.7)	Ref	–	–
	Young	228	52(22.8)	2.2	1.46-3.40	<0.001

Among host-related factors, only sex and age were significant in the univariable analysis and were fitted to the final multivariable logistic regression model (Table 3) and both sex and age of animals were identified as risk factors for the occurrence of sheep and goat pox.

**Table 3:** Multivariable logistic regression analysis of risk factors of sheep and goat pox by animal factor

Variables	Number sampled	Prevalence	Odds ratio	Confidence interval (95%CI)	*P* value
Sex					
Male	251	28(11.15%)	Ref	–	–
Female	421	76(18.05%)	1.9	1.18 3.05	0.008
Age					
Adult	444	52(11.7%)	Ref	–	–
Young	228	52(22.8%)	2.2	1.46 3.40	<0.001

### Retrospective study

**Fig. 3:**
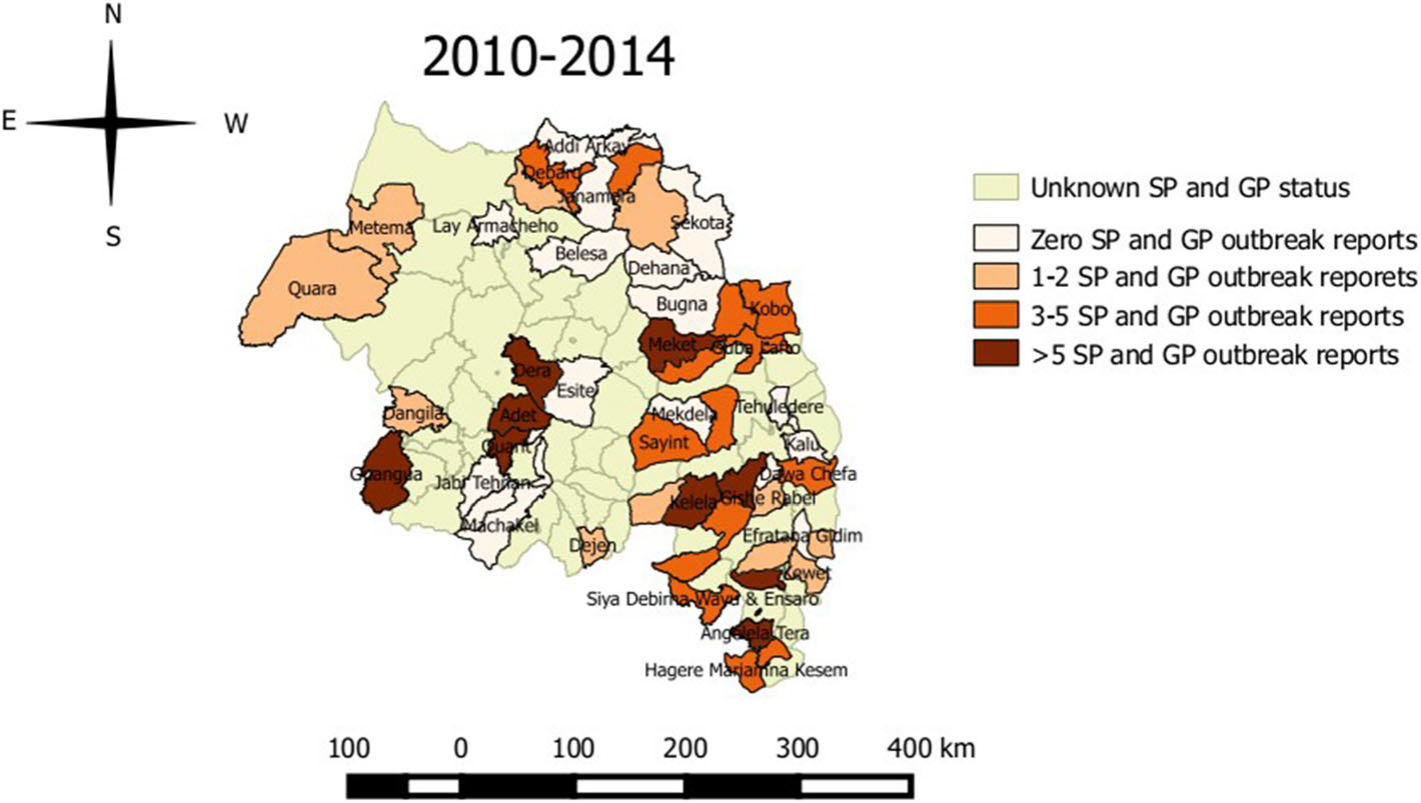
Map showing the number of SP and GP outbreak and their spatial distribution in various parts of Amhara region (sum of 2010-2014 outbreak reports)

**Fig. 4:**
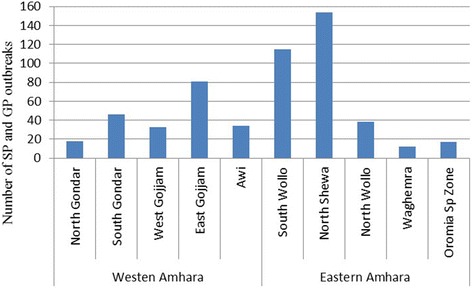
SP and GP outbreaks in Amhara Regional State from January 2010 to December 2014

Analysis of the retrospective data indicates that SP and GP outbreaks occur in all zones of the region, both in the western and eastern Amhara regions (Fig**.** 3). From January 2010 to December 2014, a total of 548 SP and GP outbreaks with 22, 888 cases and 2477 deaths were recorded in the region. Species-wise, 366 outbreaks, 12, 822 cases and 1480 deaths were in sheep, and 182 outbreaks, 10,066 cases and 997 deaths in goats. The highest outbreak was recorded in North Shoa (154) followed by South Wollo (115) and the least was recorded in Waghemra (12) within the last 5 years period (Fig**.** 4).

**Fig. 5:**
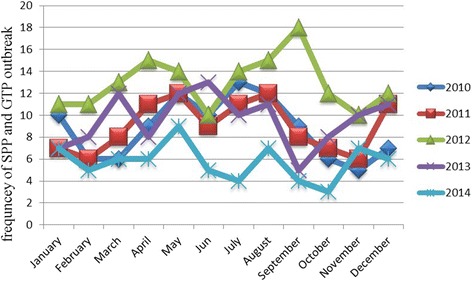
Seasonal pattern of SP and GP disease outbreaks from 2010 to 2014

As to the temporal pattern of outbreaks, the highest numbers of outbreak were reported in 2012 (155 outbreaks) followed by 2013 (115 outbreaks), and 2011 (108 outbreaks), whereas the lowest number of outbreaks (12 outbreaks) were recorded in 2014. The 5 years outbreak record indicates that the diseases occurred in all months of the year, but the highest number of outbreaks has occurred in May and August, and the lowest in February and October (Fig. 5).

Figure 4 shows the distribution of SP and GP in Amhara National Regional State. The map is based on the retrospective data obtained from MoLF database. The distribution of SP and GP in the region could be much wider, as many of the districts in the region haven’t reported the status.

## Discussion

Occurrences of sheep and goat pox in Ethiopia have been documented in World Organization for Animal Health Information Database since 1996. In the present study, we used VNT to assess whether sheep and goat in Ethiopia are exposed to sheep and goat poxviruses. Using VNT as a measure of sero-prevalence, it was found that 17% of sheep and 14% of goats were exposed to SP and/or GP viruses. The sero-prevalence report showed that both sheep and goat were equally exposed to sheep and goat pox in the study area.

The distribution of sheep and goat pox were widespread, all the study zones and districts had animals that were sero-positive for sheep and goat poxviruses. However, the sero-positivity was varied among districts. The sero-prevalence finding in this study was quite lower than the reports of Elshafie and Ali [10] and Masoud et al. [19] in Sudan and Pakistan. The difference in sero-prevalence by location could be attributed to the difference in animal movement and introduction of new animals (possibly infected). The district which showed higher sero-positivity (Fogera) is associated with extensive livestock movements for grazing and marketing activities and due to recent occurrence of the disease as an outbreak. Outbreaks of SP and GP have reported from all administrative zones of the Amhara region between 2010 and 2014 and the sero-prevalence findings in the study districts confirm the retrospective study result.

Comparison of the sero-prevalence of sheep and goat pox in different age groups of sheep and goat showed a higher seropositivity in young animals than adult animals (OR: 2.2; 95%OR: 1.46, 3.40) and the difference was highly significant (*p* < 0.001). The odds of seropositivity also showed that female (OR: 1.99; 95%CI: 1.18, 3.05; *P* = 0.008) animals were more affected than male animals. This result is in agreement with previous works reported by Elshafie and Ali [10] in Sudan and Masoud et al. [19] in Pakistan. The higher sero-prevalence in young and female animals could be explained by the low level of immunity in young and female animals associated with lambing season or poor physiological condition. In lambs and kids, the malignant form of SP and GP has been recorded as the most common type and maternal immunity provides protection only for up to 3 months (19). Here it is important to note that, due to relatively small sample size, the risk factor analyses may not have adequate power to detect differences between risk factors. Thus, care should be taken when interpreting these results.

The retrospective data indicated a difference in SP and GP cases between years and even months. Seasonality of the diseases is also reported (6, 19) that could be explained by the capability of the viruses to survive for several months in wet and cold weather, by association with lambing season and transportation of animals for marketing, Besides, the absence of regular, obligatory and constant disease reporting or underreporting is a problem in some areas of the study region. A study by Bayissa and Bereda [20] indicated, transport and communication to be the two limiting factors in disease reporting in Ethiopia. These problems could result in irregular or absence of outbreak reports for some remote health posts and district veterinary offices. If not for the low level of disease reporting, the distribution and burden of SP and GP in Ethiopia could be much more than what is documented here. The previous study by Gelaye et al. [14] indicated GP virus to be responsible for the widespread outbreaks of SP and GP in different parts of Ethiopia. In Ethiopia, a live attenuated SPPV Kenya O-180 vaccine strain (KS1-O180) is used to immunize small ruminants against sheep and goat pox and cattle against lumpy skin disease. It’s believed that sheep and goat naturally infected with sheep and goat pox viruses and recovered from the disease remain protected for lifelong (19). However, there have been repeated reports on the insufficient protection provided by the vaccine against lumpy skin disease virus in Ethiopia. This vaccination failure would likely be caused by poor vaccine handling where the availability of electricity is limited for keeping cold chain.

## Conclusion

To our knowledge, this is the first serological evidence of sheep and goat pox presence in Ethiopia. The study indicated a wide spread distribution of sheep and goat pox in Amhara region. The widespread occurrence and the high sero-positivity of sheep and goat pox observed in this study is alarming; thus, extensive studies on epidemiology, transmission, and economic impact will be necessary. Although direct and indirect animal contacts during grazing, watering, and trading are the most important transmission means in extensive production system, it is also important to explore whether there is involvement of vector in the transmission of SP and GP or not; since genetically related viruses in the genus Capripoxvirus such as Lumpy Skin Disease Virus (LSDV) are arthropod-borne [21, 22]. Considering the role of vectors in the transmission of LSDV, the possible role of vectors in the transmission of SP and GP virus should be investigated. In the context of Ethiopia where livestock movement is uncontrolled, compulsory annual mass vaccination is recommended as the best feasible, economic and viable method for the control of sheep and goat pox.

## Additional file

Additional file 1: Dataset for sheep and goat pox. (XLSX 31 kb)

## Abbreviations

CCPP: Contagious caprine pleuropneumonia
CI: Confidence interval
GP: Goat pox
LSDV: Lumpy skin disease virus
MoLF: Federal ministry of livestock and fisheries
PPR: Peste des petits ruminants
SP: Sheep pox
VNT: Virus neutralization test

## References

[1] CSA (2013): Central statistical agency Federal Democratic Republic of Ethiopia agricultural sample survey report on livestock and livestock characteristics 2012-2013, vol. (2), Addis Ababa, Ethiopia.

[2] Behnke R, Metaferia F (2011). The contribution of livestock to the Ethiopian economy – part II. IGAD LPI working paper 02-11.

[3] Asresie A, Zemedu L (2015). Contribution of livestock sector in Ethiopian economy: a review. Adv Life Sci Technol.

[4] Babiuk S, Bowden TR, Boyle DB, Wallance DB, Kitching RP (2008). Capripoxviruses: an emerging worldwide threat to sheep, goats and cattle. Transbound Emerg Dis.

[5] Rao TVS, Bandyopadhyay SK (2000). A comprehensive review of goat pox and sheep pox and their diagnosis. Anim Health Res Rev.

[6] Kitching P, Coetzer JAW, Tustin RC (2004). Sheep pox and goat pox. Infectious diseases of livestock.

[7] Bowden T, Babiuk S, Parkyn G, Copps J, Boyle D (2008). Capripox virus tissue tropism and shedding: a quantitative study in experimentally infected sheep and goats. Virology.

[8] Ahmed MM. (2012): A study on prevalence, risk factors and economic impact of sheep pox in North and South Kordofan States of the Sudan. International Symposia on Veterinary Epidemiology and Economics proceedings, ISVEE13: Proceedings of the 13th International Symposium on Veterinary Epidemiology and Economics, Belgium, Netherlands, Poster topic 9 - Surveillance and diagnostic test evaluation, p 524.

[9] Enan KA, Intisar KS, Haj MA, Hussien MO, Taha KM, Elfahal AM, Ali YH, El Hussein M (2013). Seroprevalence of two important viral diseases in small ruminants in Marawi Province Northern State, Sudan. Int J Livest Prod.

[10] Elshafie EI, Ali AS (2008). Participatory epidemiological approaches and Sero-prevalence of sheep pox in selected localities in Kassala State, Sudan. Sudan J Vetterinary Res.

[11] Davies FG, Mbugwa G (1985). The alterations in pathogenicity and immunogenicity of a Kenya sheep and goat pox virus on serial passage in bovine foetal muscle cell cultures. J Comp Pathol.

[12] Moges N, Bogale B (2012). Assessment of major animal production and health problems of livestock development in lay-Armacheho District, Northwestern Ethiopia. American-Eurasian J Sci Res.

[13] Teshome D, Derso S (2015). Prevalence of major skin diseases in ruminants and its associated risk factors at University of Gondar Veterinary Clinic, North West Ethiopia. J Vet Sci Technol.

[14] Gelaye E, Belay A, Ayelet G, Jenberie S, Yami M, Loitsch A, Tuppurainen E, Grabherr R, Diallo A, Lamien CE (2015). Capripox disease in Ethiopia: genetic differences between field isolates and vaccine strain, and implications for vaccination failure. Antivir Res.

[15] ESGPIP (Ethiopian sheep and goat productivity improvement program). technical bulletin no.29 sheep and goat pox: causes, prevention and treatment. Addis Ababa, Ethiopia; 2009.

[16] Yeruham I, Yadin H, Van Ham M, Bumbarov V, Soham A, Perl S (2007). Economic and epidemiological aspects of an outbreak of sheeppox in a dairy sheep flock. Vet Rec.

[17] Thrusfield M (2007). Veterinary epidemiology.

[18] Boshra H, Truong T, Babiuk S, Hemida MG (2015). Seroprevalence of sheep and goat pox, Peste des Petits ruminants and Rift Valley fever in Saudi Arabia. PLoS One.

[19] Masoud F, Mahmood M, Hussain I (2016). Seroepidemiology of goat pox disease in district Layyah, Punjab, Pakistan. J Vet Med Res.

[20] Bayissa B, Bereda A. Assessment of veterinary service delivery, livestock disease reporting, surveillance systems and prevention and control measures across Ethiopia/Kenya border enhanced livelihoods in southern ethiopia (ELSE) project: CIFA ethiopia/CARE ethiopia; 2009.

[21] Chihota CM, Rennie LF, Kitching RP, Mellor PS (2001). Mechanical transmission of lumpy skin disease virus by Aedes Aegypti (Diptera: Culicidae). Epidemiol Infect.

[22] Tuppurainen ES, Stoltsz WH, Troskie M, Wallace DB, Oura CA, Mellor PS, Coetzer JA, Venter EH (2011). A potential role for Ixodid (hard) tick vectors in the transmission of lumpy skin disease virus in cattle. Transbound Emerg Dis.

